# Corrigendum

**DOI:** 10.1002/jmrs.647

**Published:** 2023-01-02

Yamamoto, S., Sakamaki, F., Takahashi, G., Kondo, Y., Taguchi, N., Esashi, S., Yuji, R., Murakami, K., Osaragi, K., Tomita, K., Kamei, S., Matsumoto, T., Imai, Y. and Hasebe, T. (2022), Chest digital dynamic radiography to detect changes in human pulmonary perfusion in response to alveolar hypoxia. J Med Radiat Sci. https://doi.org/10.1002/jmrs.619

The authors would like to acknowledge the re‐use of previously published content in the creation of Figure 3 and to correctly provide attribution in that figure's caption.

The revised caption for Figure 3 should read:

Process of image analysis for pulmonary perfusion. (A) Raw data of chest radiography of participant No. 2. The image analysis software (KINOSIS 1.00; Konica Minolta, Inc., Tokyo, Japan) automatically determines the edges of the lung fields (white frame) and the region of interest (10 × 10 mm) in the left ventricle (black frame). The lung fields were separated at 5‐mm intervals, and changes in the pixel value of each block were analysed. (B) Circulation‐related pixel value changes indicate changes in the blood volume per unit volume. This schematic part is based on Tanaka R. Dynamic chest radiography: flat‐panel detector (FPD) based functional X‐ray imaging. *Radiol Phys Technol* 2016; 9: 139–53 and is adapted with permission.

The authors apologise for any inconvenience caused by this correction.

